# Effects of Different Salinity Conditions on Regulation of *ghrh-sst-gh-igf* Axis in Nile Tilapia (*Oreochromis niloticus*): Insights from Transcriptional Signature

**DOI:** 10.3390/ijms26178261

**Published:** 2025-08-26

**Authors:** Zhao Li, Pichayapa Meekuan, Ya-Xin Wang, Zhuo-Hang Feng, Shuang-Yue Luo, Zheng-Xiang Zhang, Jun Xiao, Fan Yu, Zhi-Shuai Hou

**Affiliations:** 1 China (Guangxi)-ASEAN Key Laboratory of Comprehensive Exploitation and Utilization of Aquatic Germplasm Resources, Ministry of Agriculture and Rural Affairs, Key Laboratory of Aquaculture Genetic and Breeding and Healthy Aquaculture of Guangxi, Guangxi Academy of Fishery Sciences, Nanning 530021, China; 2 Key Laboratory of Mariculture, Ministry of Education, Ocean University of China, Qingdao 266003, China; 3 Fish Feed Technology Development Center, Development of Aquaculture, Faculty of Fisheries, Kasetsart University, Chatuchak, Bangkok 10900, Thailand; 4 Fisheries College, Hunan Agricultural University, Changsha 410128, China

**Keywords:** Nile tilapia, salinity adaptation, *ghrh-sst-gh-igf* axis, aquaculture

## Abstract

Nile tilapia (*Oreochromis niloticus*) is a key species due to its rapid growth, high nutritional value, and adaptability to diverse environments. However, changes in water salinity pose significant challenges to tilapia farming. Elucidating the adaptive strategies of tilapia to fluctuating salinity environments is crucial for improving aquaculture efficiency. This study investigated the transcriptional signature of growth-hormone-releasing hormone, somatostatin, growth hormone, and insulin-like growth factor (*grhr-sst-gh-igf*) axis in Nile tilapia under different salinity conditions (0 g/L, 16 g/L, and 30 g/L). The results showed that in brackish or seawater, Nile tilapia rapidly upregulate brain *igfbp5* paralogues and their regulators (*sst5*, *sstr2*) to sustain growth-active IGF-1 signaling, while in the liver and gut, they downregulate *sstr2b*, *igfbp1/7*, and *ghrh* to reallocate energy toward osmoregulation. Physiological regulation, such as the use of ligand analogs, or genetic enhancement targeting these genes might hold promise for improving salt acclimation, which would enable profitable farming in brackish or coastal ponds and offer a simple tool for more resilient and efficient tilapia production.

## 1. Introduction

Aquaculture is essential to the global food industry, providing a significant portion of the world’s protein supply [1]. Among various aquatic species, Nile tilapia (*Oreochromis niloticus*) stands out as one of the most widely farmed fish due to its rapid growth, high nutritional value, and remarkable adaptability to diverse environmental conditions [2]. However, the tilapia farming industry faces numerous challenges, particularly from environmental fluctuations such as changes in water salinity [3]. Salinity is a critical abiotic factor affecting fish physiology, metabolism, and overall health [4,5]. Understanding how fish adapt to varying salinity conditions is crucial for enhancing their survival and growth in aquaculture.

Nile tilapia are highly adaptable to changing salinity and can survive from fresh to brackish and even salt-water after acclimation [6], which makes them ideal for aquaculture in different geographical regions. In addition, investigation of the tolerance and acclimatization of Nile tilapia to different salinity levels provides a unique opportunity to understand the underlying physiological and molecular mechanisms of these adaptations [7]. Therefore, understanding the mechanisms by which tilapia are adapt to various salinity levels might greatly contribute to developing more efficient aquaculture practices.

The growth-hormone-releasing hormone, somatostatin, growth hormone, and insulin-like growth factor (GHRH-SST-GH-IGF) axis acts as the key endocrine pathway that regulates growth, metabolism, and stress responses in fish [4,6,7,8,9]. In brief, hypothalamic GHRH stimulates pituitary GH secretion, whereas GH secretion is inhibited by SST [10]. Circulating GH then induces peripheral IGF-1 production, which in turn promotes somatic growth and drives ion-transporting organ (gill, kidney, and intestine) remodeling during salinity acclimation [11]. IGF-binding proteins (IGFBPs) fine-tune IGF bioavailability, whereas GH receptors (GHR) and IGF receptors (IGFR) mediate tissue-specific responses [6,12]. Importantly, the GHRH-SST-GH-IGF axis is conserved across vertebrates, but its gene repertoire has expanded in teleosts through whole-genome duplication, and recent functional studies have revealed that is plays pleiotropic roles in both growth and osmoregulation [13]. Investigation of the transcriptional regulation of the GHRH-SST-GH-IGF axis under varying salinity conditions is essential to revealing the adaptive mechanisms of Nile tilapia, thereby informing aquaculture practices.

Previous studies showed effects of salinity change on gene expression in fish, underscoring the importance of genes implicated in endocrinological regulations for osmoregulation [5,14,15,16]. For example, in Asian seabass (*Lates calcarifer*), the hepatic *igf1* gene is upregulated in seawater when compared to that in freshwater and brackish water [16]. However, comprehensive, multi-tissue mapping of the complete GHRH-SST-GH-IGF axis gene set under controlled salinity gradients is still lacking for Nile tilapia. Previous studies reported the GHRH-SST-GH-IGF gene repertoire in rainbow trout, and have demonstrated that paralogous genes within this pathway exhibit distinct transcriptional dynamics during salinity and pathogen challenges, and mediate energy trade-offs between growth and osmoregulation/immunomodulation [17,18]. Building on these advances, it was concluded that paralogous genes in the GHRH-SST-GH-IGF axis exhibited pleiotropic effects in osmoregulatory regulation. Although both Nile tilapia and salmonids are euryhaline fish, they have different evolutionary histories in terms of whole-genome duplications (3R of tilapia vs. 4R of salmonids, where R indicates a round of whole-genome duplication), making the conservation and functional divergence of their paralogs worth studying. Therefore, the present study quantified the transcriptional signature of the *ghrh-sst-gh-igf* axis in Nile tilapia exposed to freshwater, brackish water, and seawater conditions in order to clarify the molecular mechanisms underlying their salinity adaptation. Our results revealed that different salinity environments altered the transcriptions of the *ghrh-sst-gh-igf axis*, providing valuable insights for optimizing aquaculture efficiency through endocrine regulation.

## 2. Results

### 2.1. The Transcriptional Signature of the ghrh-sst-gh-igf Axis in the Brain Under Different Salinities

**Figure 1:**
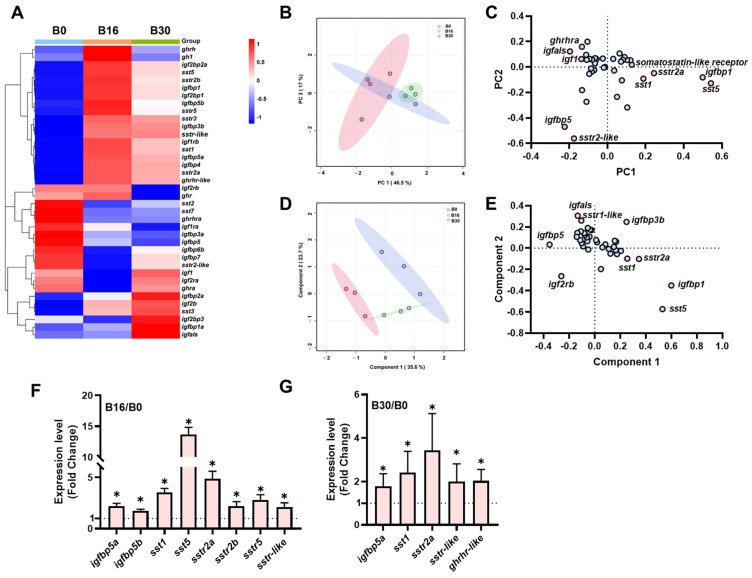
Transcriptional signature of *ghrh-sst-gh-igf* axis in brains of tilapia exposed to three salinity levels (0, 16, and 30 ppt) for 7 days. (**A**) Heatmap of *ghrh-sst-gh-igf* axis in brains. (**B**,**C**) Principal component analysis (**B**) and loading plot (**C**) of *ghrh-sst-gh-igf* axis. (**D**,**E**) Partial least squares-discriminant analysis (**D**) and loading plot (**E**) of *ghrh-sst-gh-igf* axis. In loading plot, gene(s) further away from center point (0, 0) show obvious effects on principal component analysis/partial least squares-discriminant analysis. Pink dots exert a significant contribution to both the first and second principal components, whereas blue dots manifest a comparatively attenuated contribution. (**F**,**G**) Key genes of *ghrh-sst-gh-igf* axis of B16/B0 (**F**) and B30/B0 (**G**). The dash line functions as a reference baseline, affording an intuitive means of determining whether gene expression is up- or down-regulated relative to the B0 group. Genes were selected based on principle *p* value < 0.05 according to Student’s *t*-test, “*” *p* < 0.05. B30: brains of tilapia exposed to salinity level of 30 ppt; B16: brains of tilapia exposed to salinity level of 16 ppt; B0: brains of tilapia exposed to freshwater.

The heatmap revealed distinct expression patterns of the genes within the *ghrh-sst-gh-igf* axis across different salinities (Figure 1A). Notably, significant differences in gene expression were observed between salinity levels, particularly in the 16 ppt and 30 ppt groups, when compared to the 0 ppt group. PCA indicated that the B16 group was distinctly separate from the B0 group (Figure 1B). PC1 and PC2 accounted for 48.5% and 27.2% of the total variance, respectively. Genes such as *ghrhra, igfals1,* and *sst5* exhibited high loadings on PC1 and PC2 (Figure 1C). PLS-DA further demonstrated clear separation among the different salinity treatment groups, with Component 1 and Component 2 explaining 35.6% and 23.7% of the total variance, respectively (Figure 1D). Genes including *igfbp5*, *igfbp3b*, and *igf2rb* showed high loadings on Component 1 and Component 2 (Figure 1E). The brains of tilapia in the B16 group showed upregulated expression of the *igfbp5* (*igfbp5a* and *igfbp5b*), *sst1*, *sst5*, *sstr2a*, *sstr2b*, *sstr5*, and *sstr-like* genes compared to the B0 group. Meanwhile, the *igfbp5a*, *sst1*, *sst2a*, *sstr-like*, and *ghrhr-like* genes were significantly upregulated in the B30 group relative to the B0 group (Figure 1G).

### 2.2. The Transcriptional Signature of the ghrh-sst-gh-igf Axis in the Intestine Under Different Salinities

**Figure 2:**
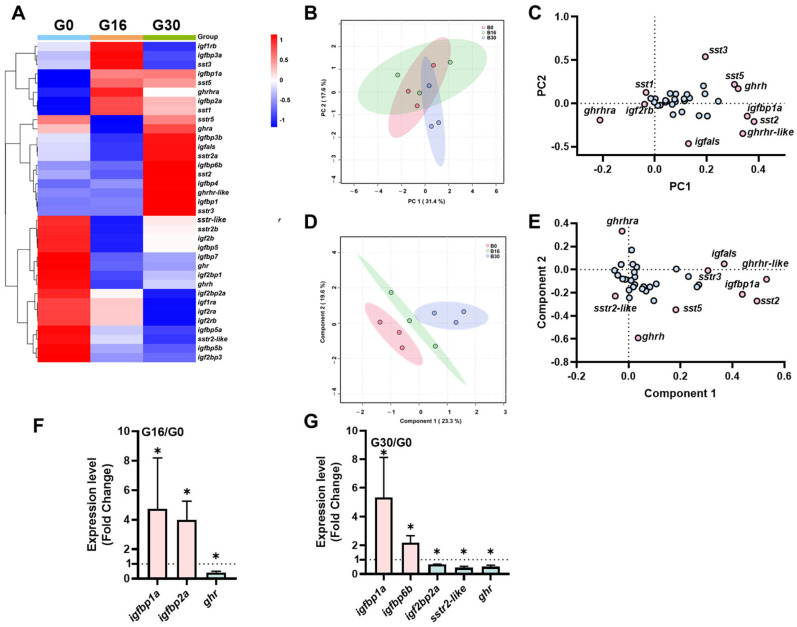
Transcriptional signature of *ghrh-sst-gh-igf* axis in intestines of tilapia exposed to three salinity levels (0, 16, and 30 ppt) for 7 days. (**A**) Heatmap of *ghrh-sst-gh-igf* axis in brains. (**B**,**C**) Principal component analysis (**B**) and loading plot (**C**) of *ghrh-sst-gh-igf* axis. (**D**,**E**) Partial least squares-discriminant analysis (**D**) and loading plot (**E**) of *ghrh-sst-gh-igf* axis. In loading plot, gene(s) further away from center point (0, 0) showed obvious effects on principal component analysis/partial least squares-discriminant analysis. Pink dots exert a significant contribution to both the first and second principal components, whereas blue dots manifest a comparatively attenuated contribution. (**F**,**G**) Key genes of *ghrh-sst-gh-igf* axis of B16/B0 (**F**) and B30/B0 (**G**). The dash line functions as a reference baseline, affording an intuitive means of determining whether gene expression is up- or down-regulated relative to the B0 group. Genes were selected based on principle *p* value < 0.05 according to Student’s *t*-test, “*” *p* < 0.05. G30: guts of tilapia exposed to salinity level of 30 ppt; G16: guts of tilapia exposed to salinity level of 16 ppt; G0: guts of tilapia exposed to freshwater.

The heatmap shows the different expression patterns of the *ghrh-sst-gh-igf* axis genes in the gut under different salinity conditions (Figure 2A). The PCA demonstrated that PC1 and PC2 accounted for 31.4% and 16.1% of the total variance, with samples forming distinct clusters based on their salinity exposure (Figure 2B). Genes such as *ghrhra, ghrhr-like,* and *sst5* exhibited high loadings on PC1 and PC2 (Figure 2C). PLS-DA revealed pronounced separation among the three salinity groups (Figure 2D). Genes including *igfbp1a*, *ghrh*, *sstr2-like*, and *ghrhr-like* were recognized as pivotal contributors to group discrimination (Figure 2E). Compared to the G0 group, genes such as *igfbp1a* and *igfbp2a* showed significant upregulation in the G16 group, while *ghr* showed significant downregulation (*p* < 0.05) (Figure 2F). Similarly, in the G30 group, genes including *igfbp1a* and *igfbp6b* were significantly upregulated, while *igf2bp2a*, *sstr2-like,* and *ghr* showed significant downregulation (*p* < 0.05) (Figure 2G).

### 2.3. The Transcriptional Signature of the ghrh-sst-gh-igf Axis in the Liver Under Different Salinities

**Figure 3:**
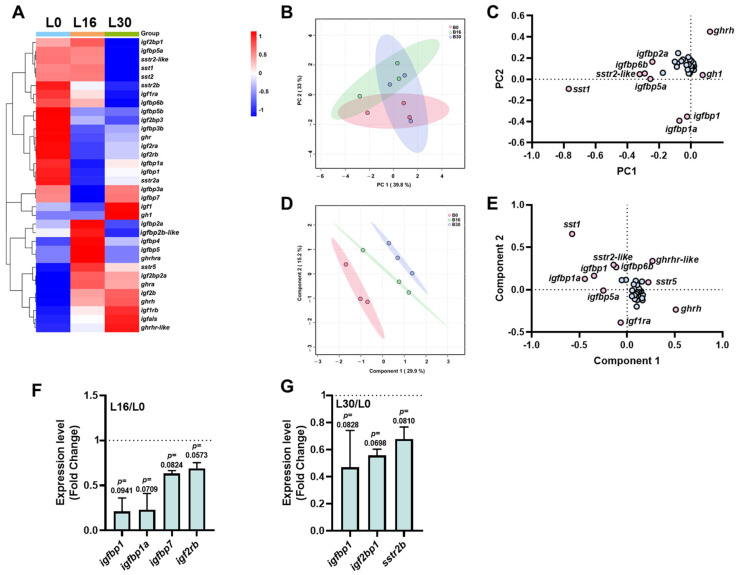
Transcriptional signature of *ghrh-sst-gh-igf* axis in livers of tilapia exposed to three salinity levels (0, 16, and 30 ppt) for 7 days. (**A**) Heatmap of *ghrh-sst-gh-igf* axis in brains. (**B**,**C**) Principal component analysis (**B**) and loading plot (**C**) of *ghrh-sst-gh-igf* axis. (**D**,**E**) Partial least squares-discriminant analysis (**D**) and loading plot (**E**) of *ghrh-sst-gh-igf* axis. In loading plot, gene(s) further away from center point (0, 0) showed obvious effects on principal component analysis/partial least squares-discriminant analysis. Pink dots exert a significant contribution to both the first and second principal components, whereas blue dots manifest a comparatively attenuated contribution. (**F**,**G**) Key genes of *ghrh-sst-gh-igf* axis of B16/B0 (**F**) and B30/B0 (**G**). The dash line functions as a reference baseline, affording an intuitive means of determining whether gene expression is up- or down-regulated relative to the B0 group. Genes were selected based on principle *p* value < 0.05 according to Student’s *t*-test. L30: livers of tilapia exposed to salinity level of 30 ppt; L16: livers of tilapia exposed to salinity level of 16 ppt; L0: livers of tilapia exposed to freshwater.

The heatmap displays the expression profiles of the *ghrh-sst-gh-igf* axis genes, revealing distinct expression patterns (Figure 3A). PCA showed that PC1 and PC2 accounted for 39.8% and 15.1% of the total variance, respectively (Figure 3B). The loading plot identified genes (*ghrh*, *igfbp1a*, and *sst1*) with significant contributions to the principal components (Figure 3C). PLS-DA was performed to discriminate between the groups, and demonstrated clear separation among the different salinity treatments (Figure 3D). Genes such as *igfbp1a*, *sst1*, and *ghrh* were recognized as pivotal contributors to group discrimination (Figure 3E). Compared to the L0 group, genes such as *igfbp1* (*p* = 0.0941), *igfbp1a* (*p* = 0.0709), *igfbp7* (*p* = 0.0824), and *igf2rb* (*p* = 0.0573) showed downregulated trend in the L16 group (Figure 2F). Similarly, in the L30 group (Figure 3G), genes including *igfbp1* (*p* = 0.0828), *igf2bp1* (*p* = 0.0698), and *sstr2b* (*p* = 0.0810) showed a trend of downregulation.

### 2.4. Correlation Analysis of ghrh-sst-gh-igf Axis Genes

**Figure 4:**
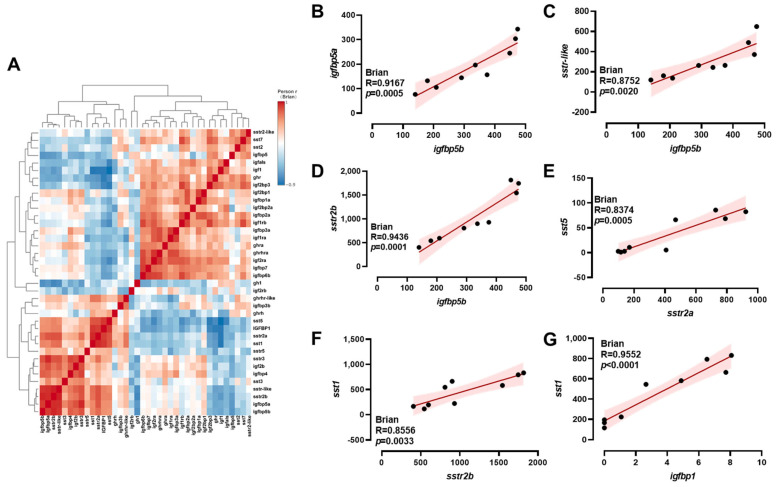
Correlation analysis of *ghrh-sst-gh-igf* axis genes in the brain. Heatmap of correlations among *ghrh-sst-gh-igf* axis genes (**A**) and Pearson correlation coefficients of two genes (**B**–**G**) in the brain. Red: positive correlations; Blue: negative correlations.

**Figure 5:**
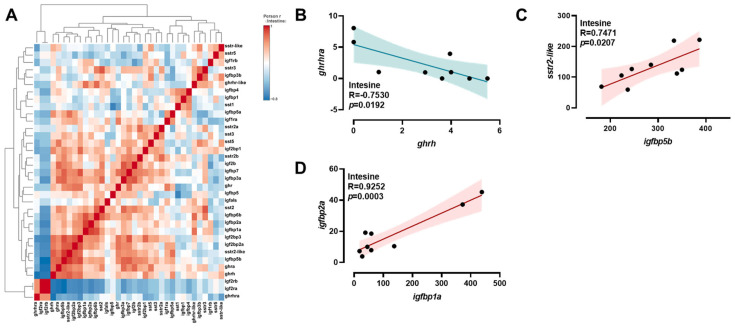
Correlation analysis of *ghrh-sst-gh-igf* axis genes in the intestine. Heatmap of correlations among *ghrh-sst-gh-igf* axis genes (**A**) and Pearson correlation coefficients of two genes (**B**–**D**) in the intestine. Red: positive correlations; Blue: negative correlations.

**Figure 6:**
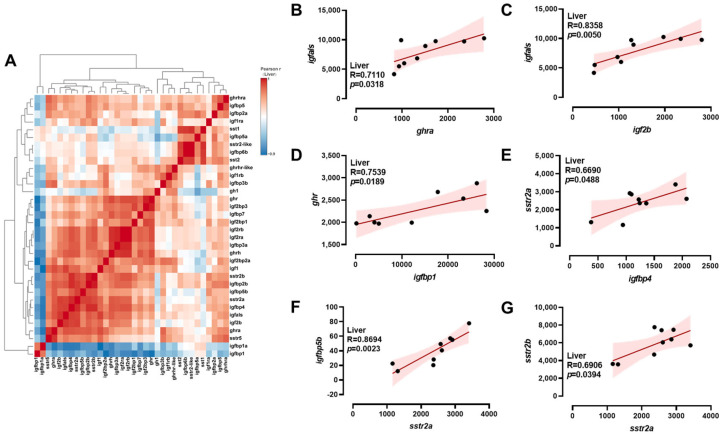
Correlation analysis of *ghrh-sst-gh-igf* axis genes in the liver. Heatmap of correlations among *ghrh-sst-gh-igf* axis genes (**A**) and Pearson correlation coefficients of two genes (**B**–**G**) in the liver. Red: positive correlations; Blue: negative correlations.

A comprehensive analysis of transcriptional correlations among *ghrh-sst-gh-igf* axis genes in the brain was conducted using a hierarchical clustering heatmap (Figure 4A). In the brain, *igfbp5b* exhibited positive correlations with *igfbp5a*, *sstr-like,* and *sstr2b* (Figure 4B–D). Meanwhile, positive correlations were observed between *sstr2a* and *sst5* (Figure 4E); *sstr2b* and *sst1* (Figure 4F); and *igfbp1* and *sst1* (Figure 4G). A comprehensive analysis of the transcriptional correlations among *ghrh-sst-gh-igf* axis genes in the intestine was conducted using hierarchical clustering heatmaps (Figure 5A). In the intestine, *ghrh* exhibited negative correlations with *ghrhra* (Figure 5B). Conversely, positive correlations were observed between *igfbp5b* and *sstr2-like*, and between *igfbp1a* and *igfbp2a* (Figure 5C,D). A comprehensive analysis of the transcriptional correlations among *ghrh-sst-gh-igf* axis genes in the liver was conducted using hierarchical clustering heatmaps (Figure 6A). In the liver, positive correlations were observed between *ghra* and *igfals*; *igf2b* and *igfals*; *igfbp1* and *ghr*; and *igfbp4* and *sstr2a* (Figure 6B–E). Additionally, *sstr2a* exhibited positive correlations with both *igfbp5b* and *sstr2b* (Figure 6F,G).

## 3. Discussion

### 3.1. Brian Transcriptional Response of ghrh-sst-gh-igf Axis Genes to Salinity Stress

Rapid adjustment of brain GH-IGF signaling is central to osmotic acclimation in tilapia. Salinity stress is a common environmental pressure that fish encounter in natural conditions, particularly in estuary, brackish, and marine environments [19]. Nile tilapia, a widely distributed and economically valuable freshwater fish species, exhibits the ability to adapt to salinity changes, enabling it to thrive in a variety of aquatic environments [2]. This study highlighted the significance of the *grhr-sst-gh-igf* axis in regulating salinity adaptation.

The brain serves as the central hub in fish for sensing and regulating salinity stress [20]. The neuroendocrine system detects changes in external salinity and modulates osmotic balance within the body through a complex network of signaling pathways [21]. Previous studies showed that changes in salinity can affect the expression patterns of genes in the *ghrh-sst-gh-igf* axis in rainbow trout (*Oncorhynchus mykiss*) [17]. In the present study, we consistently observed that the transcriptional signature of brain genes in the *ghrh-sst-gh-igf* axis was altered by environmental salinity.

IGF1 acts as an important regulator of growth and metabolism in vertebrates [22]. A recent study in teleosts revealed that IGF1 triggers salt secretion machinery duing salinity stress [23]. We observed that *igfbp5a*, *igfbp5b*, *sst5*, *sstr2*, and *ghrhr* were upregulated under high-salinity conditions. The expression changes in *ghrhr* and *sst* might directly affect GH secretion [24], thus modulating the synthesis of IGF-1 in response to increased salinity. GH promotes growth and metabolism of animals by stimulating the synthesis and release of IGF. In this study, the tilapia showed upregulated *igfbp5* subtypes in the brain. Previous studies confirmed that *igfbp5* might enhance the bioactivity of IGF-1, thus promoting cell proliferation and growth [25,26], which are essential to maintaining the growth of Nile tilapia under high salinity. Therefore, we might conclude that Nile tilapia enhance their IGF1 synthesis and bioactivity to counteract the SST-induced suppression of the GH-IGF axis in response to salinity change.

### 3.2. Divergent Roles of igfbp5a and igfbp5b in Salinity Adaptation

Igfbp-5 represents the most conserved member of the IGFBP family in mammals. Previous evidence indicates that IGFBP5 subtypes are involved in conserved roles across various aspects of teleost biology, including growth and ionic homeostasis [27]. Differential regulation of talapia’s duplicated *igfbp5* paralogues provides this species with a tunable IGF-signaling toolkit for progressive salinity challenge. Following teleost-specific whole-genome duplication, two *igfbp5* subtypes, *igfbp5a* and *igfbp5b,* were identified in Nile tilapia. Previous studies in zebrafish showed that IGFBP-5a and 5b exhibit divergent expressions and cellular actions, thus regulating IGF signaling in slightly different ways [28]. In Nile tilapia, correlation analysis showed that *igfbp5a* was positively correlated with *igfbp5b* in the brain, and both were significantly upregulated when salinity increased to 16 ppt. However, transcriptional differences between *igfbp5a* and *igfbp5b* were observed when the salinity increased to 30 ppt, with only *igfbp5a* being significantly upregulated at this salinity level. Consistent with our results, studies in zebrafish have shown that IGFBP-5a is crucial to the conditional proliferation of ionocytes in order to maintain ion homeostasis [28].

### 3.3. Intestinal Transcriptional Re-Programming Under Salinity Stress

Intestinal re-programming of the GH-IGF axis enables tilapia to balance nutrient uptake and ion regulation when external salinity rises. The intestines primarily maintain osmotic balance and nutrient supply through ion absorption and nutrient transport under salinity stress [29]. In the present study, salinity conditions of 16 ppt and 30 ppt led to upregulated *igfbp1a*, *igfbp2a*, and *igfbp6b* and downregulated *igf2bp2a*, *sstr2-like*, and *ghr* in the intestines, which have previously been highlighted for their roles in ion absorption and nutrient transport [30]. Meanwhile, correlation analysis showed that *igfbp1a* was positively correlated with *igfbp2a*. Previous studies showed that teleost *igfbp1* and *igfbp2* subtypes are closely associated with stress-related conditions such as food deprivation and infection [31,32,33]. The upregulation of *igfbp1a* and *igfbp2a* might enhance the bioactivity of IGF-1, promoting the proliferation of gut cells and nutrient absorption [34], thereby helping Nile tilapia maintain stable gut function in high-salinity environments.

An in vivo study confirmed that IGF2BP2 depletion is associated with energy metabolism [35]. The downregulation of *igf2bp2a* in our study suggested that tilapia might reduce their growth-related energy expenditure, thus diverting more energy to maintaining osmotic balance and ion regulation. Meanwhile, *igfbp6b* was significantly upregulated when salinity increased to 30 ppt. Previous studies showed that overexpression of either of *igfbp6*’s subtypes in zebrafish significantly reduces embryonic growth, indicating its role in growth inhibition [36]. Moreover, several studies in teleosts revealed that *igfbp6* subtypes were involved in food intake manipulation [27]. While *igfbp6* is linked to growth and energy allocation, studies of Atlantic salmon showed high *igfbp6* expression in the gills with dynamic expression changes during smoltification, suggesting osmoregulatory involvement [27]. Our future research will focus on exploring the pleiotropic roles of IGFBP6 in regulating growth, energy allocation, and osmoregulation.

Our results showed that under 30 ppt salinity, both intestinal *ghrh* and *sstr-like* were downregulated. The simultaneous downregulation of *ghrh* and *sstr* genes in the intestines might be part of a feedback regulatory mechanism for GH secretion [37]. Modulating *grgh* gene expression to curtail GH secretion diminishes growth-related energy expenditure. Concurrently, the downregulation of *sstr* alleviates the suppression of GH secretion, thereby preserving adequate GH levels to sustain critical physiological functions, including the complex orchestration of nutrient sensing and utilization [11,12]. This adaptive mechanism is of paramount importance for the survival and growth of Nile tilapia.

### 3.4. Hepatic Energy Homeostasis and the ghrh-sst-gh-igf-Axis

Liver-specific downregulation of axis genes constitutes a metabolic switch that preserves energy for osmoregulation while salvaging IGF signaling via GH rebound. Energy homeostasis plays a vital role in enabling organisms to adapt to environmental changes. The liver serves as an important target for metabolic regulation in Nile tilapia under salinity stress [38]. The results of this study revealed a trend of down-regulation in genes within the *ghrh-sst-gh-igf* axis, such as *igfbp1*, *igfbp7*, *igf2rb*, and *sstr2b,* under 16 ppt and 30 ppt conditions. This result aligns with prior studies suggesting that these genes may play a crucial role in regulating liver metabolism and energy supply [29,30,39,40]. For example, the decreasing trend of *igfbp1* and *igfbp7* gene expression may reduce IGF-1 bioactivity, potentially leading to decreased *igfr* expression [41], which results in metabolic disorders and inadequate energy supply in the liver. In the hypothalamus, SST plays an important role in inhibiting GH release by activating the receptors (SSTR) [22], and a previous study showed that downregulation of *sstr2b* promotes GH secretion [42]. Therefore, downregulated *sstr2b* might trigger a supplementary mechanism to restore IGF function by promotinng GH secretion, which is of great significance for the survival and growth of Nile tilapia.

## 4. Materials and Methods

### 4.1. Ethical Statement

This study was conducted following the ethical standards set forth by the Animal Research and Ethics Committee at Ocean University of China (OUC-AE-2023-358, approved on 5 July 2023). Furthermore, all experimental procedures fully complied with the guidelines for the care and use of laboratory animals as detailed in the National Institutes of Health (NIH) Publication No. 8023, revised in 1987. No endangered or protected animals were involved in this study, and the effect of gender was not considered because the juvenile tilapia were immature.

### 4.2. Experimental Design and Sample Collection

Nile tilapia (~9–11 g) were obtained from the key laboratory of comprehensive development and utilization of aquatic germplasm resources of Guangxi province, China. Nile tilapia (~100,000 individuals) were cultured in a freshwater (0 ppt) pond at the National Guangxi Nanning Tilapia Breeding Farm of the Guangxi Fishery Science Research Institute. The breeding pond covered an area of 1500 square meters with a water depth of 1.5 m. Fish were fed twice a day. Before the experiment, fish (one and half months old) were cultured in freshwater tanks (0 ppt) for 7 days at 24 ± 2 °C, under a 12 h light and 12 h dark photoperiod. Water quality was continuously monitored. Feeding was carried out twice a day at 1% body weight with a commercial diet.

Nile tilapia were randomly divided into nine 90 L tanks with three salinity groups × three replicates × 9 fish per replicate. After 7 days of acclimation in freshwater, the salinity was raised step-wise (4 ppt day^−1^) to target levels of 0 ppt (control), 16 ppt (brackish), and 30 ppt (seawater) using artificial sea salt (HaiXing sea crystal salt). Regardless of the time required to reach the target, both the 16 ppt and 30 ppt groups were held at their final salinity for a full 7 days before sampling; this post-transfer period paralleled the 7-day freshwater acclimation that all fish had undergone before the experiment. In this study, the three levels of salinity correspond to freshwater, brackish water, and marine aquaculture environments, respectively. We chose a salinity level of 16 ppt based on findings from a published paper on Nile tilapia [7], which demonstrated that this level significantly alters gene expression within the GHRH-SST-GH-IGF axis. Additionally, a salinity level of 30 ppt is representative of seawater condition [43]. Fish remained at final salinity for 7 days to mimic routine salinity transfer protocols used in tilapia hatcheries. Tanks (water volume 60 L, total volume 90 L) were stocked at 9 fish per tank with a stock density of less than 2 kg/m^3^. No mortality occurred during the transition or exposure period.

Sampling was performed at the end of the 7-day exposure period. Fish were euthanized with 200 mg L^−1^ MS-222. Two individuals were pooled into one sample to reduce individual variation for RNA-Seq analysis. The sacrificed individuals were weighed, and tissues (brain, intestine, and liver) were dissected within 2 min. Tissues were snap-frozen in liquid nitrogen and stored at −80 °C until RNA extraction.

### 4.3. RNA-Seq and Analysis

Total RNA from the brain, intestine, and liver was extracted using the TRIzol method and treated with RNase-free DNase I to eliminate genomic DNA. RNA integrity was assessed using an RNA Nano 6000 Assay Kit with the Bioanalyzer 2100 system (Agilent Technologies, Santa Clara, USA). A total of 27 libraries (3 tissues (brain, intestine, and liver) × 3 replicated samples (1 sample was conducted by pooling three individuals) × 3 treatment groups (0, 16, and 30 ppt)) were prepared using the TruSeq™ RNA Sample Prep Kit (Illumina, San Diego, USA). RNA-seq libraries were sequenced on the Illumina Novaseq platform to generate 150 bp paired-end raw reads. Clean reads for subsequent analysis were obtained from the raw reads by removing raw reads with adapters, reads containing ploy-N, and low-quality reads [44]. After that, the clean reads were aligned to the reference genome (GCA_001858045.3) and gene model annotation files using HISAT (HISAT2 v2.0.5) as the mapping tool [45], a highly efficient spliced alignment program for RNA-seq reads [45]. Differential expression analysis of genes between two groups (0 ppt vs. 16 ppt; 0 ppt vs. 30 ppt; 16 ppt vs. 30 ppt) was performed using the DEGSeq (version 1.26.0) R package (DESeq2 R package 1.20.0) with parameters set at ‘‘*p*-value” < 0.05 and |log2(fold change)| > 1. The top 30 differential expression genes were selected based on “*p*-value” ranking between the groups to plot the heatmaps (Figure S1). For the heatmaps that specifically illustrate the *ghrh-sst-gh-igf* axis, genes were pre-selected as follows: based on genomic repertoire, all the genes involved in the *ghrh-sst-gh-igf* axis were extracted from the transcriptome. Genes with an expression level of 0 were not considered.

### 4.4. Quantitative Real-Time PCR (qPCR) for Verification of RNA-Seq

Nine genes were selected for qPCR analysis to verify the reliability of the RNA-seq data (Figure S2). High-quality total RNA was used as a template for the synthesis of first-strand cDNA by using the All-In-One 5X RT MasterMix reverse transcription kit (Applied Biological Materials Inc., Richmond, BC, Canada). The primers used for qPCR are shown in Table S1. The BlasTaq^TM^ 2X qPCR masterMix kit (Applied Biological Materials Inc., Canada) was used to analyze qPCR according to the manufacturer’s instructions. For each gene and sample, three biological replicates and three technical replicates were performed simultaneously. The 2^−ΔΔCt^ method was employed to quantify the relative gene expression as described by [46]. *18sRNA* was used as a housekeeping gene.

### 4.5. Statistical Analysis

MetaboAnalyst is a widely used platform for analyzing metabolomic and transcriptomic data [47,48,49]. Following the online protocols of MetaboAnalyst (https://www.xialab.ca/tools.xhtml, accessed on 6 May 2025) and referring to prior studies [19,47,48,49,50,51,52], the normalized transcriptional data of the *ghrh-sst-gh-igf* axis were analyzed for multivariate analyses, including principal component analysis (PCA), partial least squares-discriminant analysis (PLS-DA), and heatmap visualization for pattern discovery. PCA and PLS-DA analysis showed 95% confidence regions with the loading plot. The gene expression analyses were presented as fold changes between treatment groups of 16 (30) ppt and treatment groups of 0 ppt via GraphPad Prism 8.0, with *p* < 0.05 indicating significant differences. The correlation analysis of gene expressions was evaluated by Pearson’s correlation coefficient using GraphPad Prism 8.0, with *p* < 0.05 indicating significant differences.

## 5. Conclusions

This study provides the first multi-tissue expression patten of the complete *ghrh-sst-gh-igf* axis in Nile tilapia subjected to controlled variation of salinity conditions. Integrating brain, intestine, and liver responses, we demonstrate that tilapia deploy paralogue-specific transcriptional switches. A key example is the selective upregulation of *igfbp5a* over *igfbp5b* at 30 ppt, which simultaneously sustains growth and re-programs osmoregulatory tissue. The liver-specific downregulation of *sstr2b* emerges as a novel compensatory node that boosts GH output when systemic IGF signaling is dampened, an adaptive tactic not previously documented in tilapia. These findings indicate how duplicated IGFBP genes are differentially co-opted for salinity tolerance, and offer precise endocrine markers (e.g., hepatic *sstr2b* and intestinal *igfbp1a/igfbp2a* ratios) for selective breeding or feed formulations with functional ligand analogs aimed at salt-water grow-out systems, thereby directly enhancing tilapia aquaculture efficiency under expanding brackish and marine culture scenarios.

## Data Availability

The data are contained within the article. The raw transcriptome sequencing data (PRJNA1300777) used in this study have been uploaded to NCBI.

## Supplementary Materials

The following supporting information can be downloaded at: https://www.mdpi.com/article/10.3390/ijms26178261/s1.

## References

[1] Boyd C.E., McNevin A.A., Davis R.P. (2022). The contribution of fisheries and aquaculture to the global protein supply. Food Secur..

[2] Hoseinifar S.H., Doan H.V. (2023). Tilapia Fish for Future Sustainable Aquaculture.

[3] Zimmermann S., Kiessling A., Zhang J. (2023). The future of intensive tilapia production and the circular bioeconomy without effluents: Biofloc technology, recirculation aquaculture systems, bio-RAS, partitioned aquaculture systems and integrated multitrophic aquaculture. Rev. Aquac..

[4] Bal A., Panda F., Pati S.G., Das K., Agrawal P.K., Paital B. (2021). Modulation of physiological oxidative stress and antioxidant status by abiotic factors especially salinity in aquatic organisms. Comp. Biochem. Physiol. Part C Toxicol. Pharmacol..

[5] Velasco J., Gutiérrez-Cánovas C., Botella-Cruz M., Sánchez-Fernández D., Arribas P., Carbonell J.A., Millán A., Pallarés S. (2018). Effects of salinity changes on aquatic organisms in a multiple stressor context. Philos. Trans. R. Soc. B Biol. Sci..

[6] Canosa L.F., Bertucci J.I. (2023). The effect of environmental stressors on growth in fish and its endocrine control. Front. Endocrinol..

[7] El-Leithy A.A.A., Hemeda S.A., Naby W.S.H.A.E., Nahas A.F.E., Helmy Z.A. (2019). Optimum salinity for Nile tilapia (*Oreochromis niloticus*) growth and mRNA transcripts of ion-regulation, inflammatory, stress- and immune-related genes. Fish Physiol Biochem..

[8] Cerdá-Reverter J.M., Canosa L.F. (2009). Chapter 1 Neuroendocrine Systems of the Fish Brain. Fish Physiol..

[9] Canosa L.F., Chang J.P., Peter R.E. (2007). Neuroendocrine control of growth hormone in fish. Gen. Comp. Endocrinol..

[10] Yunker W.K., Smith S., Graves C., Davis P.J., Unniappan S., Rivier J.E., Peter R.E., Chang J.P. (2003). Endogenous Hypothalamic Somatostatins Differentially Regulate Growth Hormone Secretion from Goldfish Pituitary Somatotropes in Vitro. Endocrinology.

[11] Caputo M., Pigni S., Agosti E., Daffara T., Ferrero A., Filigheddu N., Prodam F. (2021). Regulation of GH and GH Signaling by Nutrients. Cells.

[12] Montero-Hidalgo A.J., Rio-Moreno M.d., Pérez-Gómez J.M., Luque R.M., Kineman R.D. (2025). Update on regulation of GHRH and its actions on GH secretion in health and disease. Rev. Endocr. Metab. Disord..

[13] Lappin F.M., Shaw R.L., Macqueen D.J. (2016). Targeted sequencing for high-resolution evolutionary analyses following genome duplication in salmonid fish: Proof of concept for key components of the insulin-like growth factor axis. Mar. Genom..

[14] Hu W., Cao Y., Liu Q., Yuan C., Hu Z. (2024). Effect of salinity on the physiological response and transcriptome of spotted seabass (*Lateolabrax maculatus*). Mar. Pollut. Bull..

[15] Jiang Y., Yuan C., Qi M., Liu Q., Hu Z. (2022). The Effect of Salinity Stress on Enzyme Activities, Histology, and Transcriptome of Silver Carp (*Hypophthalmichthys molitrix*). Biology.

[16] Azodi M., Bahabadi M.N., Ghasemi A., Morshedi V., Mozanzadeh M.T., Shahraki R., Khademzadeh O., Hamedi S., Avizhgan S. (2021). Effects of salinity on gills’ chloride cells, stress indices, and gene expression of Asian seabass (*Lates calcarifer*, Bloch, 1790). Fish Physiol. Biochem..

[17] Xiang K., Yang Q., Liu M., Yang X., Li J., Hou Z., Wen H. (2022). Crosstalk between Growth and Osmoregulation of GHRH-SST-GH-IGF Axis in Triploid Rainbow Trout (*Oncorhynchus mykiss*). Int. J. Mol. Sci..

[18] Hou Z.-S., Xin Y.-R., Zeng C., Zhao H.-K., Tian Y., Li J.-F., Wen H. (2020). GHRH-SST-GH-IGF axis regulates crosstalk between growth and immunity in rainbow trout (*Oncorhynchus mykiss*) infected with *Vibrio anguillarum*. Fish Shellfish. Immunol..

[19] Seale A.P., Cao K., Chang R.J.A., Goodearly T.R., Malintha G.H.T., Merlo R.S., Peterson T.L., Reighard J.R. (2024). Salinity tolerance of fishes: Experimental approaches and implications for aquaculture production. Rev. Aquac..

[20] Chen J., Cai M., Zhan C. (2024). Neuronal Regulation of Feeding and Energy Metabolism: A Focus on the Hypothalamus and Brainstem. Neurosci. Bull..

[21] Verburg-van Kemenade B.M.L., Cohen N., Chadzinska M. (2017). Neuroendocrine-immune interaction: Evolutionarily conserved mechanisms that maintain allostasis in an ever-changing environment. Dev. Comp. Immunol..

[22] Veldhuis J.D., Iranmanesh A., Erickson D., Roelfsema F., Bowers C.Y. (2012). Lifetime Regulation of Growth Hormone (GH) Secretion. Handbook of Neuroendocrinology.

[23] Yan J.-J., Lee Y.-C., Tsou Y.-L., Tseng Y.-C., Hwang P.-P. (2020). Insulin-like growth factor 1 triggers salt secretion machinery in fish under acute salinity stress. J. Endocrinol..

[24] Mahmoudi R., Naderi M., Dopeikar H., Khorasaninasab S.A., Seyedalhosseini S.H. (2025). Interactive effects of triploidy induction and culture densities on growth performance and stress, immune, and metabolic responses in juvenile rainbow trout (*Oncorhynchus mykiss*). Aquac. Rep..

[25] Waters J.A., Urbano I., Robinson M., House C.D. (2022). Insulin-like growth factor binding protein 5: Diverse roles in cancer. Front. Oncol..

[26] Dai W., Bai Y., Hebda L., Zhong X., Liu J., Kao J., Duan C. (2013). Calcium deficiency-induced and TRP channel-regulated IGF1R-PI3K-Akt signaling regulates abnormal epithelial cell proliferation. Cell Death Differ..

[27] Garcia de la Serrana D., Macqueen D.J. (2018). Insulin-Like Growth Factor-Binding Proteins of Teleost Fishes. Front. Endocrinol..

[28] Allard J.B., Duan C. (2018). IGF-Binding Proteins: Why Do They Exist and Why Are There So Many?. Front. Endocrinol..

[29] Edwards S.L., Marshall W.S. (2012). Principles and Patterns of Osmoregulation and Euryhalinity in Fishes. Fish Physiology.

[30] Reinecke M., Björnsson B.T., Dickhoff W.W., McCormick S.D., Navarro I., Power D.M., Gutiérrez J. (2005). Growth hormone and insulin-like growth factors in fish: Where we are and where to go. Gen. Comp. Endocrinol..

[31] Valenzuela C.A., Zuloaga R., Mercado L., Einarsdottir I.E., Björnsson B.T., Valdés J.A., Molina A. (2018). Chronic stress inhibits growth and induces proteolytic mechanisms through two different nonoverlapping pathways in the skeletal muscle of a teleost fish. Am. J. Physiol.-Regul. Integr. Comp. Physiol..

[32] Martin S.A.M., Macqueen D.J., Boudinot P., Secombes C.J., Wang T., Castro R., Alzaid A. (2016). Cross Talk Between Growth and Immunity: Coupling of the IGF Axis to Conserved Cytokine Pathways in Rainbow Trout. Endocrinology.

[33] Peterson B.C., Small B.C. (2004). Effects of fasting on circulating IGF-binding proteins, glucose, and cortisol in channel catfish (*Ictalurus punctatus*). Domest. Anim. Endocrinol..

[34] Baxter R.C. (2023). Signaling Pathways of the Insulin-like Growth Factor Binding Proteins. Endocr. Rev..

[35] Dai N., Zhao L., Wrighting D., Krämer D., Majithia A., Wang Y., Cracan V., Borges-Rivera D., Mootha Vamsi K., Nahrendorf M. (2015). IGF2BP2/IMP2-Deficient Mice Resist Obesity through Enhanced Translation of Ucp1 mRNA and Other mRNAs Encoding Mitochondrial Proteins. Cell Metab..

[36] Wang X., Lu L., Li Y., Li M., Chen C., Feng Q., Zhang C., Duan C. (2009). Molecular and functional characterization of two distinct IGF binding protein-6 genes in zebrafish. Am. J. Physiol.-Regul. Integr. Comp. Physiol..

[37] Li W., Lin H. (2010). The endocrine regulation network of growth hormone synthesis and secretion in fish: Emphasis on the signal integration in somatotropes. Sci. China Life Sci..

[38] Robinson M.W., Harmon C., O’Farrelly C. (2016). Liver immunology and its role in inflammation and homeostasis. Cell. Mol. Immunol..

[39] Gabillard J.-C., Kamangar B.B., Montserrat N. (2006). Coordinated regulation of the GH/IGF system genes during refeeding in rainbow trout (*Oncorhynchus mykiss*). J. Endocrinol..

[40] Reindl K.M., Sheridan M.A. (2012). Peripheral regulation of the growth hormone-insulin-like growth factor system in fish and other vertebrates. Comp. Biochem. Physiol. Part A Mol. Integr. Physiol..

[41] Delafontaine P., Song Y.-H., Li Y. (2004). Expression, Regulation, and Function of IGF-1, IGF-1R, and IGF-1 Binding Proteins in Blood Vessels. Arterioscler. Thromb. Vasc. Biol..

[42] Melmed S., Liu N.-A., Ren S.-G., Chesnokova V., Zhou C., Khalafi R., Pichurin O., Ben-Shlomo A. (2013). Constitutive Somatostatin Receptor Subtype 2 Activity Attenuates GH Synthesis. Endocrinology.

[43] Su M., Feng Z., Zhong Y., Ye Z., Zhang J. (2025). Insights into salinity effect on growth of the spotted scat (*Scatophagus argus*): Exploring the optimum salinity for its culture. Aquac. Rep..

[44] Bolger A.M., Lohse M., Usadel B. (2014). Trimmomatic: A flexible trimmer for Illumina sequence data. Bioinformatics.

[45] Anders S., Pyl P.T., Huber W. (2015). HTSeq—A Python framework to work with high-throughput sequencing data. Bioinformatics.

[46] Schmittgen T.D., Livak K.J. (2008). Analyzing real-time PCR data by the comparative CT method. Nat. Protoc..

[47] Zhao H., Soufan O., Xia J., Tang R., Li L., Li D. (2019). Transcriptome and physiological analysis reveal alterations in muscle metabolisms and immune responses of grass carp (*Ctenopharyngodon idellus*) cultured at different stocking densities. Aquaculture.

[48] Chong J., Wishart D.S., Xia J. (2019). Using MetaboAnalyst 4.0 for Comprehensive and Integrative Metabolomics Data Analysis. Curr. Protoc. Bioinform..

[49] Mager S., Ludewig U. (2018). Massive Loss of DNA Methylation in Nitrogen-, but Not in Phosphorus-Deficient Zea mays Roots Is Poorly Correlated With Gene Expression Differences. Front. Plant Sci..

[50] Chong J., Xia J., Stegle O. (2018). MetaboAnalystR: An R package for flexible and reproducible analysis of metabolomics data. Bioinformatics.

[51] Liu Z., Koid A.E., Terrado R., Campbell V., Caron D.A., Heidelberg K.B. (2015). Changes in gene expression of Prymnesium parvum induced by nitrogen and phosphorus limitation. Front. Microbiol..

[52] Zhang Y., Chen K., Sloan S.A., Bennett M.L., Scholze A.R., O’Keeffe S., Phatnani H.P., Guarnieri P., Caneda C., Ruderisch N. (2014). An RNA-Sequencing Transcriptome and Splicing Database of Glia, Neurons, and Vascular Cells of the Cerebral Cortex. J. Neurosci..

