# Supplementary material for: Effects of Different Salinity Conditions on Regulation of ghrh-sst-gh-igf Axis in Nile Tilapia (Oreochromis niloticus): Insights from Transcriptional Signature
Source: Int J Mol Sci. 2025 Aug 26;26(17):8261. doi: 10.3390/ijms26178261 (PMC12428277; doi:10.3390/ijms26178261)
Supplement: Supplementary file 1 [file ijms-26-08261-s001.zip › ijms-3738916-supplementary.pdf]

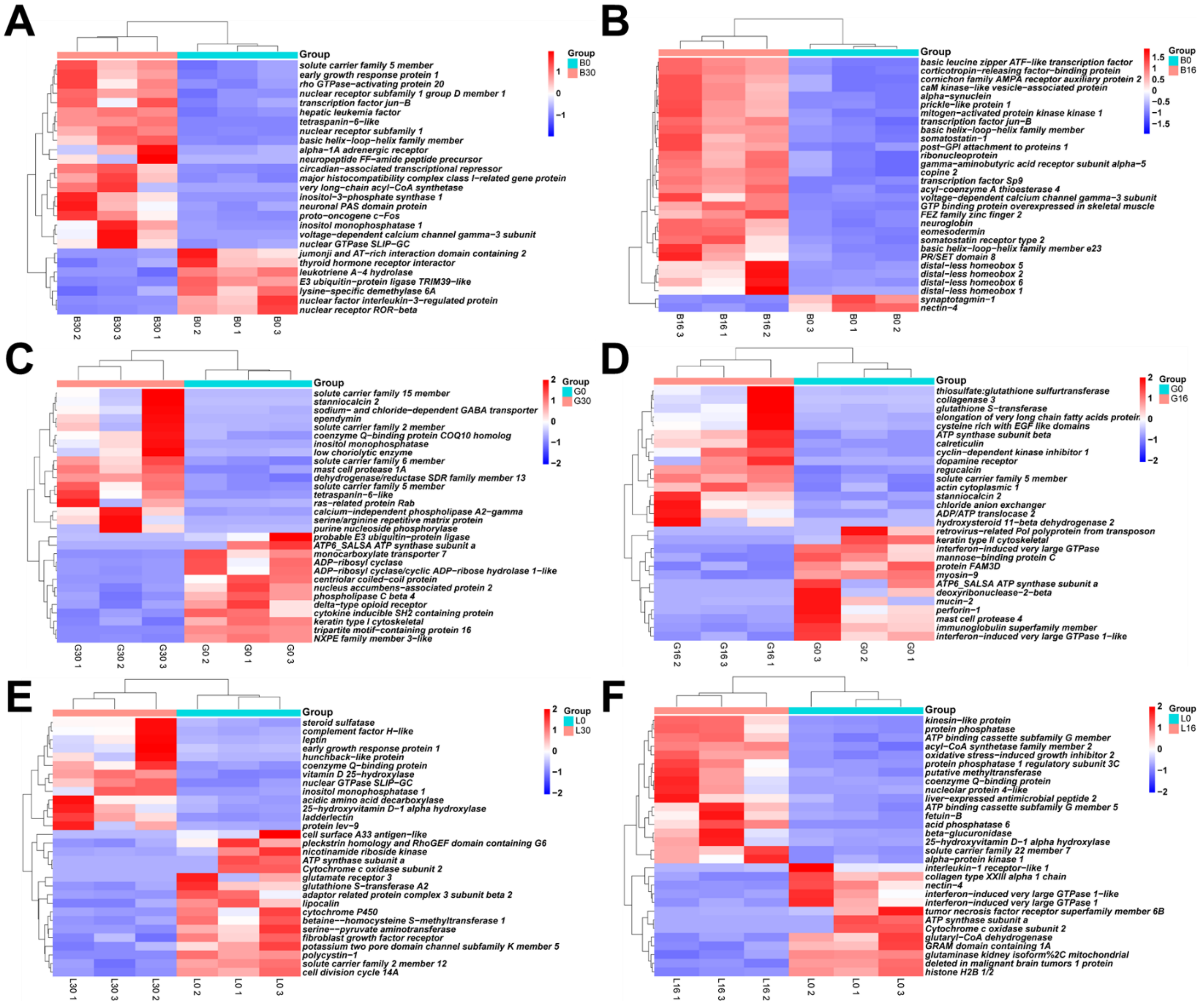

Figure S1 Heatmap of top 30 DEGs based on "*p*-value" ranking.

A: Brain 30 vs 0 ppt, B: Brain 16 vs 0 ppt, C: Intestine 30 vs 0 ppt, D: Intestine 16 vs 0 ppt,

E: Liver 30 vs 0 ppt, F: Liver 16 vs 0 ppt.

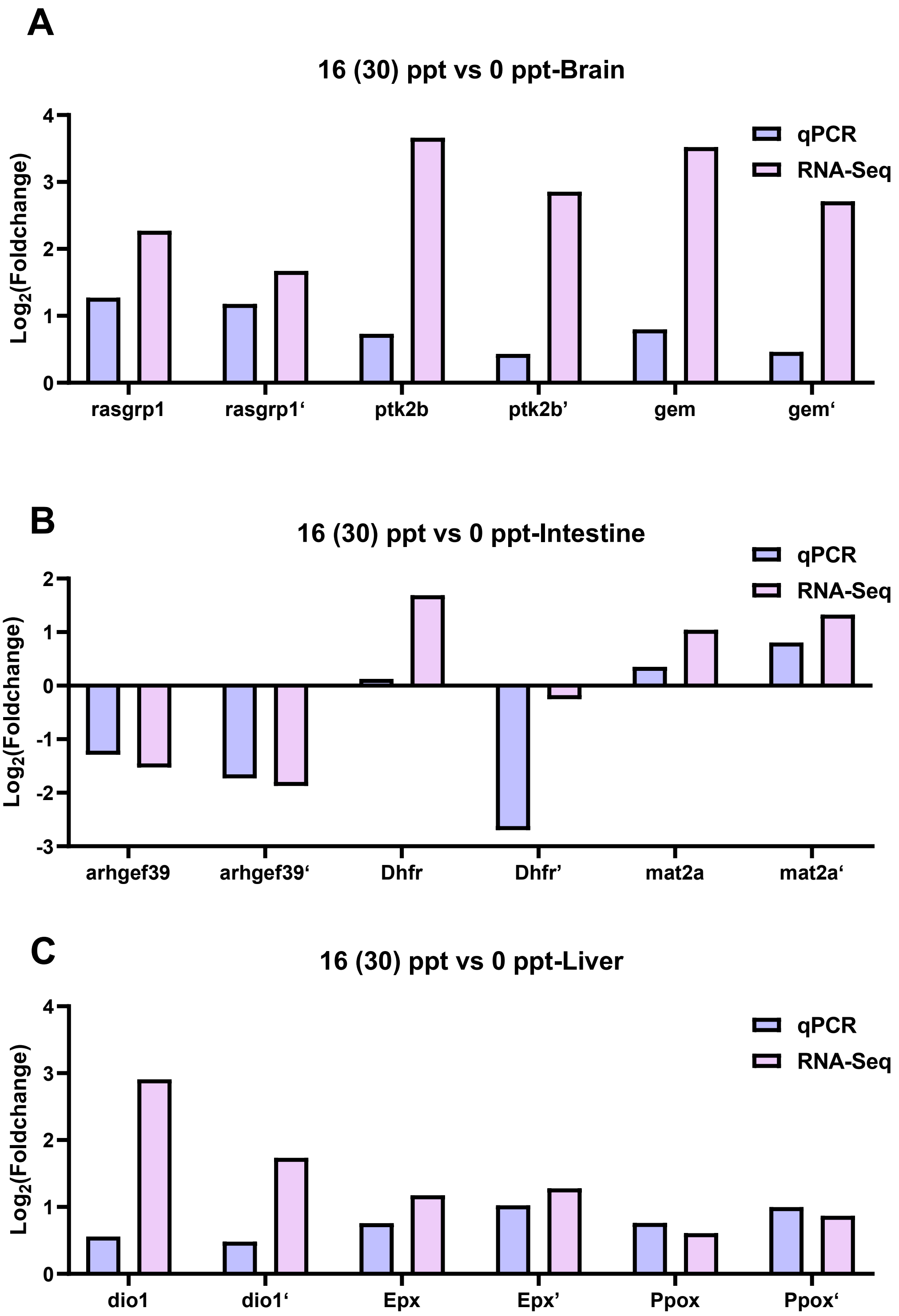

Figure S2 The qPCR gene expression validation of RNA-seq in brain (A), intestine (B) and liver (C). Note: rasgrp1 indicates group of 16 ppt vs 0 ppt, while rasgrp1' indicates group of 30 ppt vs 0 ppt.

Table S1 Primer information

Gene ID	Gene	Primers		Gene name
100699303	*rasgrp1*	Forward<br>Reverse	CCCAAGAACTGCAAAGCACC<br>TTGCACGTGTTTGCTGATGG	RAS guanyl-releasing protein 1
100691138	*ptk2b*	Forward<br>Reverse	TCGAGACTGTGCCTGAGAAG<br>GACCAAGTCCATGACGCAGT	protein tyrosine kinase 2 beta, b
100694788	*gem*	Forward<br>Reverse	TCTGAGCTCCGGATAACCCT<br>CTCGCTCACTGACACCTCTC	GTP binding protein overexpressed in skeletal muscle
100702489	*arhgef39*	Forward<br>Reverse	TTGGCCATACAAGGAGCCAG<br>GGCAGGAGTGTTGTGGCTAT	Rho guanine nucleotide exchange factor
100695790	*Dhfr*	Forward<br>Reverse	CCGTGTGCTGAACGCTATTG<br>TCACTGACGGAGTTGCTGTC	dihydrofolate reductase
100703457	*mat2a*	Forward<br>Reverse	GCCCATCCCCTCTCGATTTC<br>CCTCCCAAGGGAAGGAGTCT	methionine adenosyltransferase 2Ab
100534571	*dio1*	Forward<br>Reverse	AGCGATGTTGCCGACTTTCT<br>CAGGACACAGGGGTTCACTC	iodothyronine deiodinase 1
100697942	*Epx*	Forward<br>Reverse	CGCCAGGGAGATTCCTTGTT<br>TCAACGGCGGGATTATAGCC	myeloid-specific peroxidase
100695565	*Ppox*	Forward<br>Reverse	CGGACAGTCCCACCTTTCTC<br>CCTGCAGTCCCCTGCAAATA	protoporphyrinogen oxidase
112846812	*18sRNA*	Forward<br>Reverse	GGACACGGAAAGGATTGACAG<br>GTTCGTTATCGGAATTAACCAGAC	18S ribosomal RNA

Note: These 9 genes were selected from the DESeq2 output ($|log2FC| \geqslant 1$, *p*-vuale < 0.05) because they are enriched in GO terms for hormone signaling, oxidative-stress response and energy metabolism. Their salinity-dependent expression supports the transcriptome-derived model that RAS-MAPK activation, redox homeostasis and thyroid-hormone regulation underlie osmotic adaptation in Nile tilapia; qPCR confirmed the same directional changes (Figure S2), validating the RNA-seq data.
